# Screening for Resistance in Farmer-Preferred Cassava Cultivars from Ghana to a Mixed Infection of CBSV and UCBSV

**DOI:** 10.3390/plants9081026

**Published:** 2020-08-13

**Authors:** Wilfred Elegba, Wilhelm Gruissem, Hervé Vanderschuren

**Affiliations:** 1 Plant Biotechnology, Institute of Molecular Plant Biology, Department of Biology, ETH Zurich, 8092 Zurich, Switzerland; wgruissem@ethz.ch; 2 Biotechnology and Nuclear Agriculture Research Institute, GAEC, P. O. Box LG 80 Legon, Accra, Ghana; 3 Institute of Biotechnology, National Chung Hsing University, Taichung City 402, Taiwan; 4 Plant Genetics, TERRA Research and Teaching Centre, Gembloux Agro BioTech, University of Liège, 5030 Gembloux, Belgium; 5 Laboratory of Tropical Crop Improvement, Division of Crop Biotechnics, KU Leuven, B-3001 Leuven, Belgium

**Keywords:** cassava brown streak disease, resistance screening, quantitative reverse transcription PCR, top graft-inoculation, virus detection, cassava germplasm, surveillance, pre-emptive strategies

## Abstract

Cassava brown streak disease (CBSD) caused by the *Cassava brown streak virus* (CBSV) and *Ugandan cassava brown streak virus* (UCBSV) is a threat to cassava production in Africa. The potential spread of CBSD into West Africa is a cause for concern, therefore screening for resistance in farmer-preferred genotypes is crucial for effective control and management. We multiplied a selection of eleven cassava cultivars grown by farmers in Ghana to test their response to a mixed infection of CBSV (TAZ-DES-01) and UCBSV (TAZ-DES-02) isolates using a stringent top-cleft graft inoculation method. Virus titers were quantified in the inoculated scions and cuttings propagated from the inoculated scions to assess virus accumulation and recovery. All cultivars were susceptible to the mixed infection although their response and symptom development varied. In the propagated infected scions, CBSV accumulated at higher titers in leaves of eight of the eleven cultivars. Visual scoring of storage roots from six-month-old virus-inoculated plants revealed the absence of CBSD-associated necrosis symptoms and detectable titers of CBSVs in the cultivar, IFAD. Although all eleven cultivars supported the replication of CBSV and UCBSV in their leaves, the absence of virus replication and CBSD-associated symptoms in the roots of some cultivars could be used as criteria to rapidly advance durable CBSD tolerance using breeding and genetic engineering approaches.

## 1. Introduction

In tropical and semi-tropical regions of the world, cassava (*Manihot esculenta* Crantz) is an important source of carbohydrate in the diets of nearly 800 million people, one-third of whom live in sub-Saharan Africa (SSA) [1,2]. Cassava production is severely constrained by viral diseases, mainly cassava mosaic disease (CMD) and cassava brown streak disease (CBSD) in SSA [3]. Geographically, CMD is widespread across all cassava-growing regions in SSA while CBSD is prevalent in low- to mid-altitude regions of Eastern and Central Africa [3,4,5,6]. Economically, estimated annual yield losses attributable to both CMD and CBSD has been reported to exceed USD 1 billion [2,3]. Although both viral diseases reduce root yield in susceptible varieties, necrotic lesions caused by CBSD further exacerbate economic losses as they make storage roots unfit for consumption and processing [7,8,9].

CBSD is caused by *Cassava brown streak virus* (CBSV) and *Ugandan cassava brown streak virus* (UCBSV), both of which are (+) sense single-strand RNA (ssRNA) viruses of the genus *Ipomovirus* (family *Potyviridae*) [9,10,11,12,13]. CBSD is transmitted over short distances by the whitefly vector, *Bemisia tabacci* (Gennadius) [14,15], while long-distance spread can occur through the transport of infected planting material [5,16]. The co-occurrence of CBSV and UCBSV species (referred to as CBSVs) has been reported in disease endemic and epidemic zones [17,18,19]. The common occurrence of mixed CBSV and UCBSV infections in the field [3,20] has prompted the research community to implement mitigation strategies that rely on resistance to both species.

Past and current efforts to control the incidence of CBSD in Eastern Africa have focused on screening for resistance or tolerance in both cultivated and wild species of cassava and on the introgression of CBSD resistance and tolerance into farmer-preferred cultivars [8,21,22]. Several varieties such as “Kiroba”, a back-cross derivative known as “Kaleso” in Kenya or ”Namikonga” in Tanzania [9] or clones MM 06/0082, MM 06/0123, MM 06/0128 and MM96/0876 have been shown to exhibit tolerance to CBSD through one or a combination of mechanisms such as restricted virus replication, reduced virus accumulation and movement as well as limited symptom expression [23,24,25,26]. Further investigation of the cassava–CBSV pathosystem has led to the identification of two breeding lines, KBH 2006/18 and KBH 2006/2, which show resistance to mixed infection of CBSV and UCBSV isolates by restricting virus movement from the vascular tissues to the mesophyll cells [27,28]. The integration of these genotypes into local farming systems and breeding programs has extended the sources of tolerance against CBSD in the field [23,26]. The recent identification of seven cassava lines in the South American cassava germplasm with high resistance to CBSD infection [29], if confirmed under field conditions, will further expand the genetic basis for introgressing CBSD resistance into farmer-preferred cassava cultivars.

Several methods (both vector and non-vector based) have been developed to screen for CBSD resistance in cassava under field and greenhouse conditions. The use of whiteflies to inoculate plants with CBSV and UCSBV isolates has been successful albeit with limited efficiency [13,14,15,25]. Side-grafting [30] as well as bud grafting methods [20,27] have enabled large-scale screening of cassava plants with limited number of CBSVs-infected plants. However, both methods have lower infection rates compared to the top grafting method, which usually achieves 100% infection rates [27,31,32]. Although the recent construction of an infectious UCBSV clone opens new opportunities to develop robust inoculation methods [33], graft inoculation methods have so far been most effective for the transmission of CBSV and UCBSV to non-infected cassava plants [20,27,31].

Notwithstanding progress in the control and management of CBSD, cultivars with natural CBSD tolerance or resistance have been mostly developed for deployment in Eastern Africa [23,24,25]. West Africa accounts for almost 50% of cassava production in Africa, with Nigeria and Ghana contributing the major portion of this production [34]. Although CBSD has not yet been reported in West Africa, comprehensive and timely screening of farmers’ fields is key to prevent CBSD pandemics in West African cassava-growing regions. The presence of CBSD in cassava fields in Burundi and Democratic Republic of Congo indicates a westward drift of the disease into areas previously considered as CBSD-free. Furthermore, the recent increase in *B. tabaci* populations in Eastern and Central Africa, which might have played a role in CBSD outbreaks in mid-altitude regions [3,35], is a cause for concern. Therefore, there is a need to implement pre-emptive strategies in West Africa in order to prevent the rapid dissemination of CBSD in regions where cassava is a major staple and industry crop.

The aim of the present study was to assess the resistance of eleven cassava cultivars preferred by farmers in Ghana to a mixed infection of CBSV (TAZ-DES-01) and UCBSV (TAZ-DES-02) [GenBank Accession numbers JN091565.1 and KF878103.1]. In-depth characterization of leaf and root CBSD symptom development, virus replication as well as the impact of vegetative propagation on viral disease transmission is reported.

## 2. Results

### 2.1. No Detection of CBSVs in Selected Field-Grown Cassava Cultivars in Ghana

Molecular screening of leaf material from eleven field-grown cassava cultivars obtained from the BNARI germplasm collection in Ghana (Table S2) and leaf material collected from farmers’ fields in Ghana (Figure S1) was carried out in order to determine their phytosanitary status (Figure S2). Leaf material from selected cassava cultivars displayed typical CMD but no CBSD symptoms. We first screened the cultivars and leaf material from farmers’ fields for CBSVs using degenerate primers that detect the coat protein of CBSVs in the three main phylogenetic clades [6,13,18] (Table S1, Figure S3). Leaf samples of the cultivars from the BNARI germplasm collection were also screened for cassava mosaic geminiviruses (CMGs) (Figure S4) with two commonly-used generic primer pairs [36,37]. We did not detect CBSVs in all analyzed leaf samples (Figure S1), which was consistent with the absence of CBSD-associated symptoms in the plants from which leaves were collected. However, we detected ACMV and EACMV species that are present in Ghana in several of the leaf samples collected from the selected BNARI cultivars (Figure S4).

Together, our results indicate that the eleven selected cassava cultivars from Ghana obtained from the BNARI germplasm collection were free of CBSVs and that CBSVs are currently absent from the surveyed farmers’ fields. However, extension of molecular screening to cassava fields across cassava-growing regions in Ghana will be important to confirm the absence of CBSVs and inform CBSD prevention strategies.

### 2.2. Susceptibility of Selected Farmer-Preferred Cassava Cultivars to Mixed Infection of CBSV (TAZ-DES-01) and UCBSV (TAZ-DES-02)

The top-cleft graft method has proven to be the most stringent method to inoculate cassava with CBSVs as demonstrated by the reported 100% transmission rates for CBSV and UCBSV [27,28,31,32]. The eleven cassava varieties collected from the cassava germplasm available at BNARI (Table S2) represent farmer-preferred cassava cultivars popular in the selected cassava producing regions of Ghana. They were propagated in the greenhouse at ETH Zurich to multiply scions for grafting. To ensure the absence of CBSV (TAZ-DES-01) and UCBSV (TAZ-DES-02) in the selected Ghanaian cassava cultivars, we performed reverse-transcription-polymerase chain reaction (RT-PCR) using specific primers that detect the coat protein of the two virus isolates (Table S1). CBSV (TAZ-DES-01) and UCBSV (TAZ-DES-02) were not detected in the selected Ghanaian cassava cultivars.

To test the response of the selected farmer-preferred cultivars to CBSD, we used Ebwanateraka cassava rootstocks carrying a mixed infection of CBSV (TAZ-DES-01) and UCBSV (TAZ-DES-02). We previously showed that Ebwanateraka rootstocks support high levels of CBSV (TAZ-DES-01) and UCBSV (TAZ-DES-02) replication [27,31,32]. The mixed infection of CBSV (TAZ-DES-01) and UCBSV (TAZ-DES-02) cause rapid development of visible foliar symptoms and accumulation of detectable levels of CBSV and UCBSV in several CBSD-susceptible cultivars [27,28,31,32].

**Figure 1:**
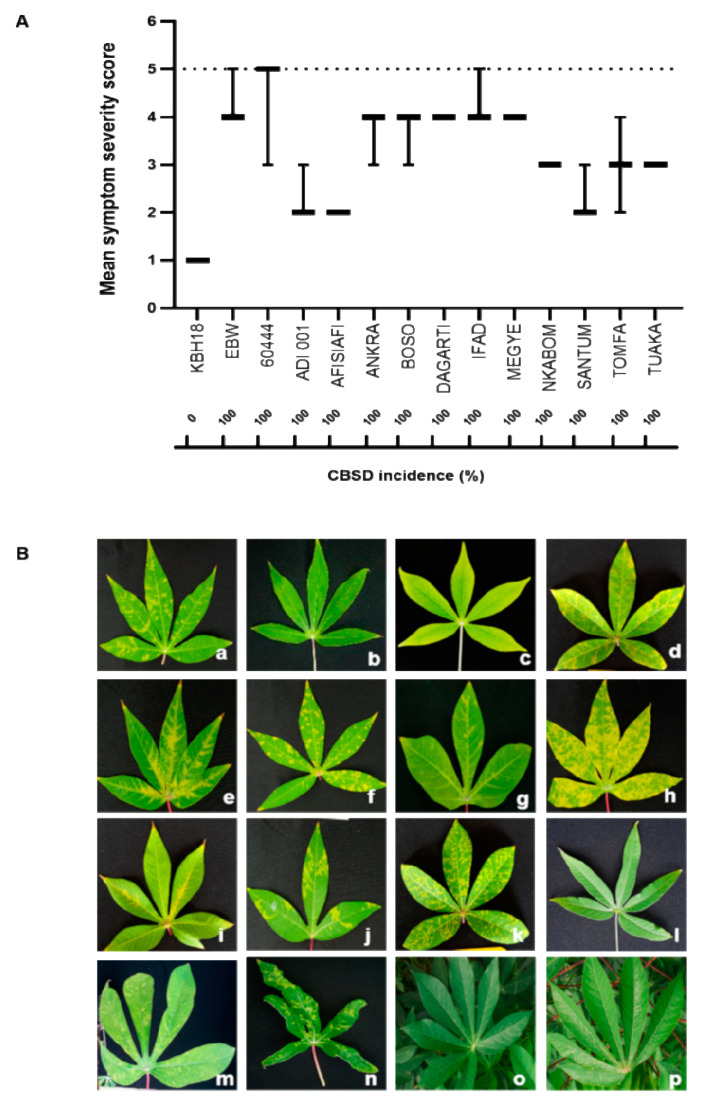
Cassava brown streak disease (CBSD) incidence, severity and symptom expression in leaves of Ghanaian cassava cultivars at 12 weeks post grafting. (**A**) CBSD incidence and mean symptom severity scores in Ghanaian cassava genotypes. (**B**) CBSD symptom expression in leaves of (a) Dagarti (b) Santum (c) Afisiafi (d) Bosomnsia (e) IFAD (f) Megyewontem (g) Tomfa (h) Ankra (i) ADI 001 (j) Nkabom (k) Tuaka (l) KBH 2006/18 (m) Ebwanateraka and (n) 60444. No symptoms were found in non-infected leaves of (o) Tuaka and (p) Ankra. CBSD incidence and mean symptom severity scores are based on three biological replicates for each cultivar. CBSD symptom expression in leaves of Ghanaian cassava cultivars was assessed using a 5-point scoring scale where 1= no visible CBSD symptoms and 5 = severe foliar symptoms and plant die-back [40,41,42].

A 100% infection incidence (Figure 1A) with typical CBSD symptoms were found in all eleven graft-challenged Ghanaian cassava cultivars (Figure 1B), although variation in symptom severity could be observed (Table S2). Consistent with earlier results [27], we did not detect any incidence of CBSD in the elite breeding line KBH 2006/18. All three replicates remained symptom-free throughout the 12-week screening period. The cultivar Afisiafi had the lowest mean CBSD leaf symptom severity (2.0), while the cultivar IFAD displayed the highest mean CBSD leaf symptom severity (4.3), similar to those observed for the two susceptible controls, Ebwanateraka and 60444 (Figure 1A). The cassava genotype Afisiafi is the improved CMD-resistant variety TMS 30572 that was released in Ghana in 1993 [38] and that is widely cultivated by farmers in Ghana. Although high CBSD incidence has been observed in most of the CMD-resistant cultivars deployed over the last decade [39], our results suggest that Afisiafi could be classified as tolerant to CBSD based on its ability to restrict symptom expression and severity under the top-graft inoculation of mixed CBSV (TAZ-DES-01) and UCBSV (TAZ-DES-02) infection [25].

### 2.3. UCBSV (TAZ-DES-02) Accumulates to Higher Levels than CBSV (TAZ-DES-01) in Infected Scions and Stem Cuttings Propagated from Scions

**Figure 2:**
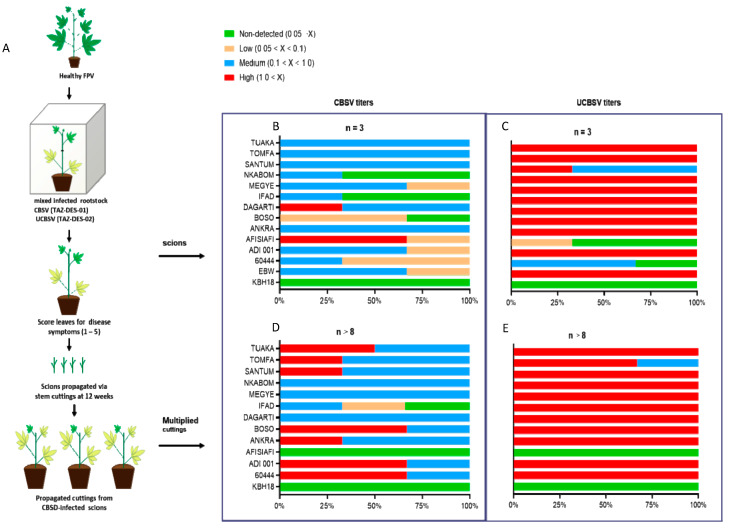
*Cassava brown streak virus* (CBSV) (TAZ-DES-01) and *Ugandan cassava brown streak virus* (UCBSV) (TAZ-DES-02) titers in scions and cuttings multiplied from scions at 12 weeks. (**A**) Stringent approach for CBSD screening of Ghanaian cassava cultivars. (**B**) CBSV (TAZ-DES-01) and (**C**) UCBSV titers in scions at 12 weeks post grafting (wpg) (n = 3) and cuttings multiplied from (**D**) CBSD (TAZ-DES-01)- and (**E**) UCBSD (TAZ-DES-02)-infected scions at 12 weeks post multiplication (wpm) (n ≥ 8). CBSV (TAZ-DES-01) and UCBSV (TAZ-DES-02) titers quantitated relative to MePP2A gene. Cuttings made from scions of cultivar Ebwanateraka failed to sprout. X = titers of CBSV (TAZ-DES-01) or UCBSV (TAZ-DES-02) relative to the PP2A gene standard. FPV = farmer-preferred variety.

In order to determine the accumulation levels of CBSV (TAZ-DES-01) and UCBSV (TAZ-DES-02) in the selected cassava cultivars, we measured viral titers in top-grafted scions as well as in cuttings propagated from inoculated scions using RT-qPCR (Figure 2A). Both CBSV (TAZ-DES-01) and UCBSV (TAZ-DES-02) accumulated in leaves from all selected cultivars, although there were differences in virus titers among cultivars. In all cultivars except Afisiafi, UCBSV (TAZ-DES-02) accumulated at higher levels compared to CBSV (TAZ-DES-01) (Figure 2B and 2C). Similar observations of higher UCBSV titers in scions of cassava genotype 60444 have been previously reported [27,31]. Under field conditions, an increase in UCBSV titers was reported in both CBSD susceptible and tolerant cultivars between three to seven months after planting [25]. CBSD symptom expression did not correlate significantly (r = −0.002, P = 0.9) with relative virus titer of CBSVs in Ghanaian cassava cultivars (Figure S5)**.** For example, even though Afisiafi had the lowest mean CBSD incident and leaf symptom severity (Figure 1), it accumulated higher levels of CBSV compared to IFAD or both susceptible varieties, Ebwanateraka and 60444 (Figure 2B). Notably, in Dagarti and Megyewontem severe CBSD leaf symptoms corresponded with high virus titers in leaves.

Virus disease management strategies in cassava have exploited the phenomenon of reversion, which is the production of virus symptom-free plants from infected parent plants [43]. Reversion has been considered a component of resistance and thus cultivars that display reversion after CBSD infection could serve as sources of tolerance against CBSD. In light of this background, we multiplied infected parental scions and evaluated the cuttings for CBSD symptoms and virus accumulation. In the propagated infected cuttings, CBSV(TAZ-DES-01) accumulated at higher titers in the leaves of eight out of eleven Ghanaian cultivars screened, although this increase was only significant in Bosomnsia (P = 0.03) (Figure 2D; Table S3A and 3B). In contrast, UCBSV (TAZ-DES-02) titers were significantly lower in propagated cuttings when compared to titers in the parental scions in three cultivars, ADI 001 (P = 0.001), Ankra (P = 0.01) and Nkabom (P = 0.001) (Figure 2E; Table S4A and 4B). Except in Bosomnsia, Santum, Tomfa, and Tuaka, which accumulated higher levels of UCBSV (TAZ-DES-02) titers, all other cultivars had lower levels of UCBSV in the propagated cuttings.

CBSV is reportedly more aggressive than UCBSV [6,19,42], although the response to either or both viruses is highly variable and dependent on the virulence of the virus isolate and varies between cassava genotypes [25,42]. In our challenge of Ghanaian cassava cultivars, we observed the accumulation of higher levels of CBSV (TAZ-DES-01) compared to UCBSV (TAZ-DES-02) in propagated infected cuttings, which supports earlier reports [6,9]. In cassava, the response to infection by CBSVs is displayed mainly in four forms: genotypes that have low virus load with restricted symptom expression, genotypes that have low virus load with relatively severe symptoms, genotypes that have high virus loads but show relatively mild symptoms, and genotypes that display severe symptoms with high virus load [25]. In contrast, there was a reduction in UCBSV (TAZ-DES-02) titers in leaves of propagated infected cuttings of Ghanaian genotypes. Similar declines in UCBSV viral loads in selected East African genotypes with progression of the growing season have been previously reported [25]. Therefore, adequate assessment of CBSD resistance in cassava requires using both CBSV and UCBSV isolates, alone or in combination. Our results indicate that all eleven Ghanaian cassava cultivars are susceptible to CBSV (TAZ-DES-01) and UCBSV (TAZ-DES-02) isolates, although the severity of symptoms and virus titers vary between genotypes.

### 2.4. CBSD Decreases Viability of Propagated Stem Cuttings in Ghanaian Cassava Cultivars

To evaluate the effect of CBSD on the viability of clonal stem cuttings propagated from infected scions of the selected Ghanaian cultivars, we multiplied a minimum of nine stem cuttings per cultivar (three nodes per cutting) from CBSD-infected scions and grew them under greenhouse conditions. Compared to CBSD non-infected cuttings, the survival rates of sprouted cuttings were reduced in all eleven Ghanaian cassava cultivars after CBSD infection. Specifically, less than 50% of cuttings propagated from infected scions failed to sprout except in Bosomnsia, Megyewontem and Nkabom, which had a higher percentage of cuttings that sprouted. For example, in Afisiafi, which was classified as tolerant based on its ability to restrict CBSD symptom expression and severity, the accumulation of high levels of CBSV titers in the inoculated scions might explain the reduced survival rates of Afisiafi cuttings. Comparatively, between 78 to 100% of cuttings propagated from non-infected Ghanaian cultivars sprouted by 12 weeks in the greenhouse (Table 1).

It has been previously reported that endemic CBSV isolates are associated with severe symptoms and significant reduction in the sprouting of cuttings compared to milder epidemic isolates [9,42]. The isolates, CBSV (TAZ-DES 01) and UCBSV (TAZ-DES-02) used in this study also reduced the survival rate of cuttings in CBSD-susceptible cultivars Ebwanateraka and 60444 [27]. CBSD infections are known to affect growing buds and sprouting of stem cuttings, particularly in susceptible cassava varieties [9,44]. In our infections of Ghanaian cassava cultivars with CBSVs, we recorded a significant (P < 0.0001) reduction in sprouting and regeneration of infected stem cuttings at 12 weeks (Table 1; Table S5A and 5B). This result confirms the impact of CBSD on cassava regeneration and survival, although other factors such as length, number of nodes per cutting, stem age and lignification of stems can also affect the quality of cassava planting material and subsequent sprouting of cuttings [45]. 

### 2.5. Accumulation of CBSVs in Storage Roots of Ghanaian Cassava Cultivars

**Figure 3:**
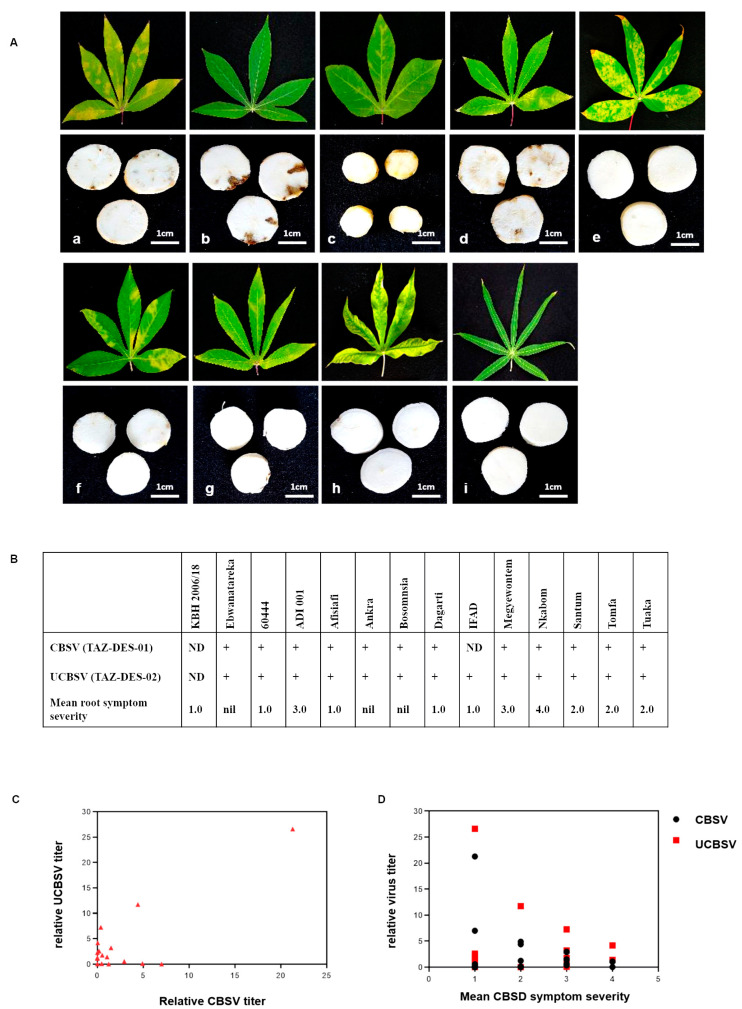
CBSD root necrosis and mean symptom severity in storage roots of Ghanaian cassava cultivars from 6-month-old plants. (**A**) Examples of leaves and storage roots from CBSD-affected plants. (a) Megyewontem (b) Nkabom (c) Tomfa (d) ADI 001 (e) IFAD (f) Tuaka (g) Afisiafi (h) 60444 (i) KBH 2006/18 = CBSD-resistant control plant. (**B**) Detection (presence (+) or not detected (ND)) of CBSV (TAZ-DES-01) and UCBSV (TAZ-DES-02) in leaves and mean symptom severity scores in storage roots (n = 3 biological samples). CBSD symptom expression in storage roots of Ghanaian cassava cultivars was assessed using a 5-point scoring scale where 1= no symptoms, 2 = less than 5% of storage root necrosis, 3 = 5–10 % necrosis, 4 = 10–25% necrosis and 5 = more than 25% necrosis [24]; nil = no storage roots produced. (**C**) Relative titers of CBSV (TAZ-DES-01) correlates significantly (r = 0.8, P < 0.001) with UCBSV (TAZ-DES-02) titers in storage roots (**D**) Necrosis symptom severity in storage roots does not correlate with either CBSV(r = −0.18, P = 0.4) or UCBSV (r = −0.06, P = 0.8) titers (Pearson correlation).

To analyze the accumulation of CBSV (TAZ-DES-01) and UCBSV (TAZ-DES-02) in storage roots of the cassava cultivars, stem cuttings were propagated from CBSD-infected scions and grown for 6 months under greenhouse conditions in order to produce storage roots. The storage roots were harvested and visually assessed for typical CBSD root necrosis symptoms. The eleven cultivars graft-challenged with rootstocks carrying a mixed infection of CBSV (TAZ-DES-01) and UCBSV (TAZ-DES-02) were subsequently propagated by stem cuttings (except Ebwanateraka, for which cuttings did not sprout; Table 1). Eight cultivars produced storage roots and typical CBSD root necrosis was observed in seven genotypes; Megyewontem, Nkabom, ADI 001, Tomfa, Tuaka, Santum and Dagarti (Figure 3). Only the storage roots of IFAD and Afisiafi did not develop brown necrotic symptoms at 6 months after planting (Figure 3A).

Using a previously described scoring scale [24], mean CBSD root symptom severity ranged from a score of 1.0 in Afisiafi, IFAD and Dagarti to a score of 4.0 in Nkabom (Figure 3B). No necrosis was seen in the CBSD-susceptible variety 60444 at six months after planting. The appearance of root necrosis is cultivar dependent and could take more than six months to appear in susceptible varieties after CBSD foliar symptoms have developed [8]. It should be noted that two CBSD-infected cultivars, Ankra and Bosomnsia, failed to form storage roots in the greenhouse after six months. Although both cultivars produce storage roots in the field [46], screening of cultivars for resistance in the greenhouse is constrained by planting pot volume, which could prevent storage root formation in Ankra and Bosomnsia.

To further confirm accumulation of CBSV and UCBSV in storage roots, we quantitated virus titers using RT-qPCR [32]. We detected both CBSV (TAZ-DES-01) and UCBSV (TAZ-DES-02) accumulation in the storage roots of all eleven cultivars except for IFAD, in which only UCBSV (TAZ-DES-02) could be detected (Figure S6). Neither CBSV (TAZ-DES-01) nor UCBSV (TAZ-DES-02) was detected in storage roots of the CBSD-resistant elite breeding line KBH 2006/18 at six months post-multiplication of cuttings from infected scions. Interestingly IFAD, which had no detectable levels of CBSV (TAZ-DES-01), also did not show necrotic symptoms in storage roots of cuttings grown for six months post multiplication of cuttings from infected scions. Although the susceptible variety 60444 had the highest accumulation of CBSV and UCBV titers in storage roots, we also did not observe visible necrotic symptoms at six months (Figure 3B; Figure S6). Our observation confirms earlier work that corky necrotic symptoms developed in storage roots of 60444 only after seven months post-CBSD infection [31]. In Afisiafi and Dagarti, we detected CBSV and UCBSV accumulation in storage roots in the absence of root necrosis. This suggests that both cultivars are able to restrict CBSD symptom expression but not virus replication in storage roots at this growth stage. Similar to leaves, we detected UCBSV (TAZ-DES-02) at higher titers than CBSV (TAZ-DES-01) in the storage roots of the Ghanaian cultivars (r = 0.8, P < 0.0001) (Figure 3C). However, mean CBSD symptom severity in storage roots did not correlate with CBSV (r = −0.18, P = 0.4) or UCBSV titers (r = −0.06, P = 0.8) (Figure 3D). Variation in cultivar response to CBSD infection has been observed in different cassava genotypes both under field and greenhouse conditions [9,13,25]. In CBSD-susceptible cultivars in particular, symptoms correlate with virus titers of either CBSV (TAZ-DES-01) or UCBSV (TAZ-DES-02), while this relationship does not hold true in genotypes displaying some level of CBSD tolerance or resistance [25].

## 3. Discussion

CBSD is an important constraint on the production of cassava in several regions of Africa [3]. Based on lessons learned in East Africa where less attention was given to CBSD until it reached epidemic levels several decades after the first report in Tanzania [3,47,48], it has become necessary to take pre-emptive measures to prevent the spread and limit the impact of CBSD in West Africa, a major cassava-producing zone in Africa. Conventional breeding and selection for resistance to CBSD in cassava germplasm (both wild and cultivated) have yielded hybrids (46106/27 and 4763/16) and clones with adequate resistance to CBSD [21,49,50]. For example, CBSD resistance in the hybrid 46106/27, which is locally known as “Kaleso” in Kenya or “Namikonga” in Tanzania has been extensively used in breeding programs and is being cultivated by farmers [9]. In addition, several CBSD-tolerant cassava clones have been identified and their resistance has been studied [27,28]. Some of these CBSD-tolerant clones have been integrated into local farming systems [5,9]. However, identification of broad-spectrum resistance remains challenging and investigation of additional gene pools can be promising to offer new sources of broad-spectrum CBSD resistance [29]. Moreover, characterization of CBSD resistance or tolerance has so far been performed with cassava cultivars popular in regions where CBSD is endemic [25,42]. However, information about the CBSD susceptibility of farmer-preferred cultivars from regions where CBSV has not yet been reported is key to the implementation of pre-emptive measures and mitigation strategies.

Characterization of tolerance or susceptibility to CBSD in cassava cultivars based on foliar symptoms alone tends to underestimate infection levels because symptom expression is influenced by a number of factors including environmental conditions, genotype, plant age and viral isolates [9]. The development of observable foliar symptoms and accumulation of high levels of CBSV and UCBSV in tissues facilitates rapid and robust screening of cassava genotypes. Therefore, we challenged the Ghanaian cassava cultivars by grafting them on rootstocks carrying a mixed infection of CBSV (TAZ-DES-01) and UCBSV (TAZ-DES-02) isolates under controlled greenhouse conditions. We found important variations in CBSD leaf symptoms among the cultivars, suggesting that CBSD symptoms in leaves are genotype-dependent. To address this variation in symptoms, we quantitated virus titers of CBSVs in leaves of the selected cultivars at 12 weeks post-grafting using an established protocol [27,31,32]. As previously observed in the genotype 60444, we also found a much higher accumulation of UCBSV in both leaves and storage roots of the selected Ghanaian cassava varieties [27,31]. In contrast, other studies have reported accumulation of higher titers of CBSV than UCBSV in stem cuttings [20,25,42]. Since all eleven selected Ghanaian cultivars displayed CBSD leaf symptoms and supported the replication of CBSVs used in the present study, we conclude that they are susceptible to CBSD. In six-month-old storage roots of the CBSD-infected Ghanaian cultivars, typical root necrosis was only observed in seven out of eleven cultivars. Although we did not identify strong CBSD resistance or tolerance in the selected Ghanaian cassava cultivars, the absence of necrotic symptoms in the six-month-old storage roots of Afisiafi and IFAD indicates the ability to restrict symptoms during early storage root growth, although it is possible root necrosis can occur during maturity [8]. However, the poor sprouting of Afisiafi from cuttings of CBSD-infected scions with high CBSV titers in the greenhouse needs to be further investigated to determine if stem cuttings from mature CBSD-infected Afisiafi plants show similar poor propagation ability. While our observations in Afisiafi and IFAD should be confirmed through the entire growth cycle and with different CBSV and UCBSV isolates, the absence of necrotic symptoms after CBSD infection remains a trait of interest when robust CBSV resistance or immunity is not available. Promising genotypes that support the replication of CBSVs, but are free of root symptoms have already been developed in breeding programs [5,51].

In addition, the absence of CBSVs especially CBSV (TAZ-DES-01) and UCBSV (TAZ-DES-02) isolates in leaf material from the farmers’ fields surveyed in four cassava-producing regions in Ghana is important because it will inform CBSD prevention strategies. The implementation of prevention strategies in a coordinated manner among the cassava community can limit the spread of CBSD into West Africa.

## 4. Materials and Methods

### 4.1. Sample Collection and Plant Material

Cuttings from field-grown plants of eleven farmer-preferred cassava cultivars, Ankra, Afisiafi, ADI 001, Bosomnsia, Dagarti, IFAD, Megyewontem, Nkabom, Santum, Tuaka and Tomfa were collected from the cassava germplasm collection of the Biotechnology and Nuclear Agriculture Research Institute (BNARI), Ghana. These cuttings were established in the greenhouse at ETH Zurich, Switzerland (27 °C, 16 h light, and 60% humidity) and subsequently multiplied by stem cuttings. In addition, to survey the presence of CBSV in Ghana, eighty cassava leaf samples were collected from farmers’ fields in four cassava-growing regions (Central region (05°41′ N, 00°34′ W, elevation 45 m; 05°35′ N, 00°35′ W, elevation 115 m), Eastern region (06°17′ N, 00°27′ W, elevation 33 m), Ashanti region (06°08′ N, 01°25′ W, elevation 90 m; 06°11′ N, 01°28′ W, elevation 146 m; 06°42′ N, 01°31′ W, elevation 280 m) and Greater Accra region (05°45′ N, 00°17′ W, elevation 95 m; 05°40′ N, 00°12′ W, elevation 55 m)). Twenty samples were collected from the Greater Accra region, twenty samples from the Central region, ten samples from the Eastern region and thirty samples from the Ashanti region. Samples were stored at −80 °C until processed.

### 4.2. Virus Isolates and Inoculation

The virus isolates used in this study were CBSV (TAZ-DES-01; GenBank Accession number KF878104.1) and UCBSV (TAZ-DES-02; GenBank Accession number KF878103.1). Both isolates were maintained in cassava genotype Ebwanateraka under greenhouse conditions at 27 °C and 60% relative humidity. Using a previously established top-grafting method [32] virus-free cassava stem cuttings were grafted onto rootstocks of Ebwanateraka carrying both CBSV (TAZ-DES-01) and UCBSV (TAZ-DES-02) virus isolates. Cassava variety 60444 and the CBSD-resistant elite breeding line KBH 2006/18 were included as positive and negative controls, respectively. All stem cuttings were maintained under greenhouse conditions as stated above.

### 4.3. CBSD Symptom Scoring

Leaves of CBSD challenged plants were visually assessed and scored for CBSD symptoms 12 weeks post grafting using a scoring scale where 1 = no visible CBSD symptoms, 2 = mild foliar symptoms on some leaves, 3 = pronounced foliar symptoms but no die-back, 4 = pronounced foliar symptoms with some die-back of terminal branches, and 5 = severe foliar symptoms and plant die-back [40,41,42]. A detrimental effect of CBSD on cassava is the necrotic lesions induced in storage roots, which causes major economic losses [7,8,9]. To assess CBSD-associated root necrosis in storage roots, graft-challenged plants were left to grow for 6 months before harvest. Roots were cut into slices approximately 1 cm in thickness with a razor blade and scored for CBSD-associated necrosis symptoms using a scoring scale previously described [24].

### 4.4. RNA Isolation and Sample Preparation

Total RNA was isolated from 1 g of cassava leaves using the modified cetyltrimethyl ammonium bromide (CTAB) method [52]. First strand cDNA was synthesized by pre-treating 1 µg of RNA with 1 µL of DNase I (ThermoFisher Scientific, Vilnius, Lithuania). The prepared RNA was used as template for cDNA synthesis using a RevertAid First Strand cDNA synthesis kit (ThermoFisher, Waltham, Massachusetts, USA) according to the manufacturer’s protocol. Specifically, 1 µL of random hexamer primer mix was added to the prepared RNA and incubated at 65 °C for 5 min. The mixture was kept on ice and 4 µL of 5X reaction buffer, 1 µL of RiboLock RNNase inhibitor (20 U/ µL), 2 µL of 10mM dNTP mix and 1 µL of RevertAid M-MuLV RT (200 U/ µL) was added to make a total reaction volume of 20 µL. For RNA extraction, leaf material collected from farmers’ fields were pooled into six sample groups for screening by RT-PCR as follows; leaf samples collected from the Eastern region (10 samples) pooled into one group, Greater Accra region (20 samples) pooled into 1 group, Central region (20 samples) pooled into 2 groups and Ashanti region (30 samples) pooled into 2 groups. For graft-challenged Ghanaian cassava cultivars, approximately 2 g of leaf material collected from the top, middle and bottom of scions was pooled (6 g in total) and used for RNA extraction.

### 4.5. Detection and Quantitation of Virus Titers

For detection of CBSV and UCBSV, cDNA was diluted in a ratio of 1:3 (1 part of cDNA to 3 parts of water). A total volume of 4 µL of the cDNA template dilution mixture was used for RT-PCR. PCR thermal cycle conditions used were as follows: Initial denaturation for 20 s at 95 °C, 40 cycles of denaturation for 3 s at 95 °C, annealing for 15 s at 60 °C and extension for 30 s at 72 °C. PCR products were resolved on 1% *w/v* agarose gel with images recorded using a Gel iX20 imager gel documentation system (INTAS science imaging system, Goettingen, Germany) to confirm the presence or absence of cassava brown streak viruses. The selected eleven Ghanaian cassava cultivars were screened for CBSVs prior to CBSD resistance challenge using degenerate primers designed to detect the coat protein of CBSVs including CBSV (TAZ-DES-01) and UCBSV (TAZ-DES-02) viral isolates [27]. Similarly, leaf material collected from farmers’ fields in four cassava-growing regions in Ghana was screened for CBSVs using the same pair of degenerate primers.

For quantitation of virus titers in leaf and storage roots of Ghanaian cultivars after CBSD graft-challenge, we performed RT-qPCR with a 7500 Fast Real-Time PCR System (Applied Biosystems, Waltham, Massachusetts, USA) using the ABI 7000 sequence detection system (SDS) for analysis. The RT-qPCR reaction mix consisted of 4 µL of cDNA template, 10 µL SYBR^®^ Green Master Mix (Applied Biosystems, ThermoFisher Scientific), 1 µL each of forward and reverse primers (1 µM final concentration) and 4 µL of distilled water in a total volume of 20 µL. For each cultivar, we analyzed samples from three individual plants (3 biological replicates) with two technical replicates each using primers specific for CBSV (TAZ-DES-01) and UCBSV (TAZ-DES-02) viral isolates [27,28] (Table S1). For storage roots, RNA was extracted from three storage roots per individual plant (pooled as one biological replicate). Amplification of cassava gene PP2A was included as internal control [27,31,32]. For detection of cassava mosaic geminiviruses in field-grown cassava cultivars and leaf samples collected from famers’ fields, we used two previously published primer pairs specific for the coat protein of both ACMV and EACMV species [37,53] or the intergenic region of ACMV [36].

## 5. Conclusions

Our screening of a panel of farmer-preferred cassava varieties in Ghana for CBSD resistance or susceptibility is alarming because all are susceptible to infection with CBSVs. However, the identification and characterization of locally preferred cassava cultivars that display reduced symptoms and virus titers could help in designing mitigation strategies that take farmer and consumer preferences into account until CBSD-resistant cultivars adapted to Ghanaian agrosystems and that meet consumer preferences become available. The absence of CBSVs, particularly CBSV (TAZ-DES-01) and UCBSV (TAZ-DES-02) isolates, in field-grown cassava cultivars in Ghana is encouraging for cassava production at this time. Nonetheless, our results should also encourage extensive and continuous surveillance of CBSVs in farmers’ fields across all cassava-producing regions in Ghana.

In addition, continued screening and characterization of cassava germplasm for responses to CBSD infection is important to identify genotypes with CBSD tolerance or resistance traits (restricted symptom expression, restriction of viral replication or accumulation). The deployment of such tolerant or resistant cultivars can effectively control the spread and reduce losses associated with CBSD infection. It is worth noting that the time and challenge associated with conventional breeding of cassava for resistance to CBSD should also encourage other approaches. Implementation of transgenic technologies in farmer-preferred varieties can complement conventional breeding and speed up the development of durable resistance against CBSD in Africa [54,55].

## Figures and Tables

**Table 1:** Survival rate of cuttings propagated from CBSD-infected Ghanaian cassava genotypes at 12 weeks old.

Genotype	Status	Control		CBSV-Inoculated		Mean CBSD Leaf Symptom Severity
		No. of Sprouted Cuttings/no. of Cuttings Made	Survival Rate (%)	No. of Sprouted Cuttings/no. of Cuttings Made	Survival Rate (%)	
KBH 2006/18	Released variety	9/9	100	11/27	41	1.0
Ebwanateraka	Released variety	7/9	78	0/15	0	-
60444	Released variety	8/8	100	3/21	14	4.3
ADI 001	Landrace	9/9	100	10/36	28	2.3
Afisiafi	Released variety	9/9	100	3/15	20	2.0
Ankra	Landrace	8/9	89	4/17	24	3.7
Bosomnsia	Landrace	8/9	89	8/9	89	3.7
Dagarti	Released variety	8/9	89	6/25	24	4.0
IFAD	Released variety	8/9	89	4/16	25	4.3
Megyewontem	Landrace	8/10	80	15/20	75	4.0
Nkabom	Released variety	9/9	100	16/27	59	3.0
Santum	Released variety	10/10	100	6/28	21	2.3
Tomfa	Released variety	9/9	100	3/13	23	3.0
Tuaka	Landrace	9/9	100	4/25	16	3.0

(-) = none of the propagated infected cuttings sprouted.

## Supplementary Materials

The following are available online at https://www.mdpi.com/2223-7747/9/8/1026/s1. Table S1: Primer sequences used for CBSV and UCBSV detection by qPCR (upper half) and RT-PCR (bottom half) in leaves and storage roots of Ghanaian cassava cultivars. Figure S1: Map showing location of cassava fields from which leaf samples were collected for CBSD screening. Figure S2: PCR screening of eleven selected field-grown cassava cultivars in Ghana. Figure S3: Homology of primers used for RT-PCR screening of cassava cultivars and leaf material collected from farmers’ fields in Ghana. Figure S4: PCR screening of eleven selected cassava cultivars grown in Ghana for cassava mosaic geminiviruses. Table S2: Top-cleft graft transmission of CBSV and UCBSV to farmer-preferred Ghanaian cassava cultivars at 12 weeks. Figure S5: Pearson correlation between CBSD symptom severity and relative CBSV titers in leaves of Ghanaian cassava genotypes. Table S3A: Two-way ANOVA comparing CBSV titers in parental scions and cuttings propagated from scions of Ghanaian cassava cultivars. Table S3B: Šidák’s multiple comparisons test of CBSV titers in parental scions and cuttings propagated from scions of Ghanaian cassava cultivars. Table S4A: Two-way ANOVA comparing titers of UCBSV in parental scions and cuttings propagated from scions of Ghanaian cassava cultivars. Table S4B: Šidák’s multiple comparisons test of UCBSV titers in parental scions and cuttings propagated from scions of Ghanaian cassava cultivars. Table S5A: Two-way ANOVA comparing survival rate of cuttings propagated from CBSD-infected Ghanaian cassava cultivars at 12 weeks old. Table S5B: Šidák’s multiple comparisons test of survival rates of cuttings propagated from CBSD-infected Ghanaian cassava cultivars at 12 weeks old. Figure S6: RT-qPCR quantitation of CBSV and UCBSV in roots of Ghanaian cassava genotypes (6-month-old plants).
