# Supplementary material for: Screening for Resistance in Farmer-Preferred Cassava Cultivars from Ghana to a Mixed Infection of CBSV and UCBSV
Source: Plants (Basel). 2020 Aug 13;9(8):1026. doi: 10.3390/plants9081026 (PMC7465500; doi:10.3390/plants9081026)
Supplement: Supplementary file 1 [file plants-09-01026-s001.pdf]

Table S1. Primer sequences used for CBSV and UCBSV detection by qPCR (upper half), RT-PCR (middle) and PCR (bottom half) in leaves and storage roots of Ghanaian cassava cultivars

Primer name	Primer sequence	Target sequence	Detection test
TAZ:DES:01_CBSV CP<br>TAZ01_CP3	FP-5′ -TTGACTCAACCAGAGAAGTTTATGGG -3′<br>RP- 5′- GCGGTTTGAAGCTCTTCCGA -3′	CBSV coat protein	qPCR
TAZ-DES-02_UCBSV _HAM1h-like	FP- 5′- AAGCCTGACGGGTGTGGTTGT-3′<br>RP- 5′- GCCTTCTTCTCCTCTTGCTTCACC-3′	UCBSV coat protein	
MePP2A	FP- 5′- TGCAAGGCTCACACTTTCATC-3′<br>RP- 5′- CTGAGCGTAAAGCAGGGAAG-3′	PP2A transcript	
CP3F<br>CP3R	FP- 5′- CCTCCATCWCATGCTATAGACA-3<br>RP- 5′- GGATATGGAGAAAGRKCTCC-3	CBSV coat protein	RT-PCR
UCBSVF<br>UCBSVR	FP- 5 ′- CCATTTGAGGCTAGGAGATTG- 3′<br>RP- 5′- ACTTCCCCATCATCTGGTTCTC- 3′	UCBSV coat protein	
MePP2A	gFP -5′- CGCTGTGGAAATATGGCATCA– 3′<br>gRP -5′- CTGGCTCAAACTGCAGGATCAA– 3′	PP2A genomic	
UV-AL1/F<br>ARO/R	FP- 5 ′- CCATTTGAGGCTAGGAGATTG- 3′<br>RP- 5′- GCTCGTATGTATCCTCTAAGGCCTG-3′	ACMV intergenic region and AV2 gene	PCR
JSP001/JSP002	FP- 5′ - ATGTCGAAGCGACCAGGAGAT<br>RP- 5′- TGTTTATTAATTGCCAATACT 3	ACMV/EACMV coat protein	

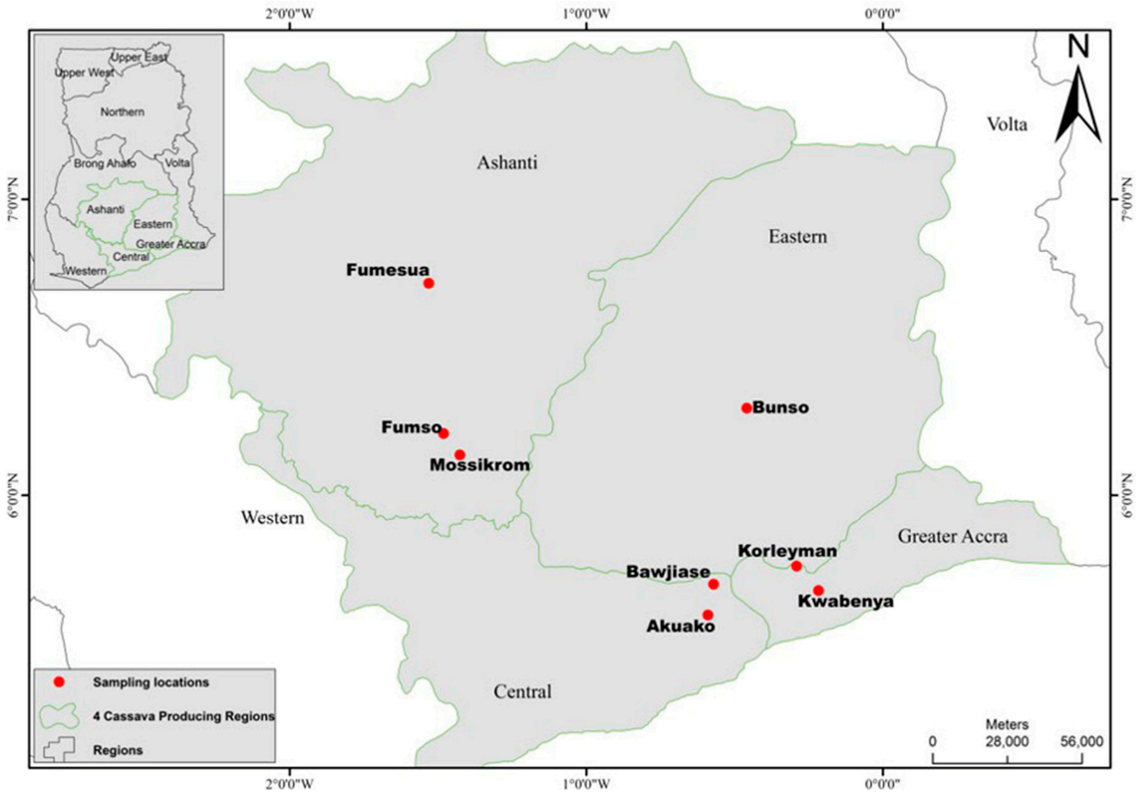

Figure S1. Map showing location of cassava fields from which leaf samples were collected for CBSD screening.

Bawjiase and Akuako = Central Region); Bunso = Eastern Region; Mossikrom, Fumso, Fumesua = Ashanti Region and Kwabenya, Korleyman = Greater Accra Region.

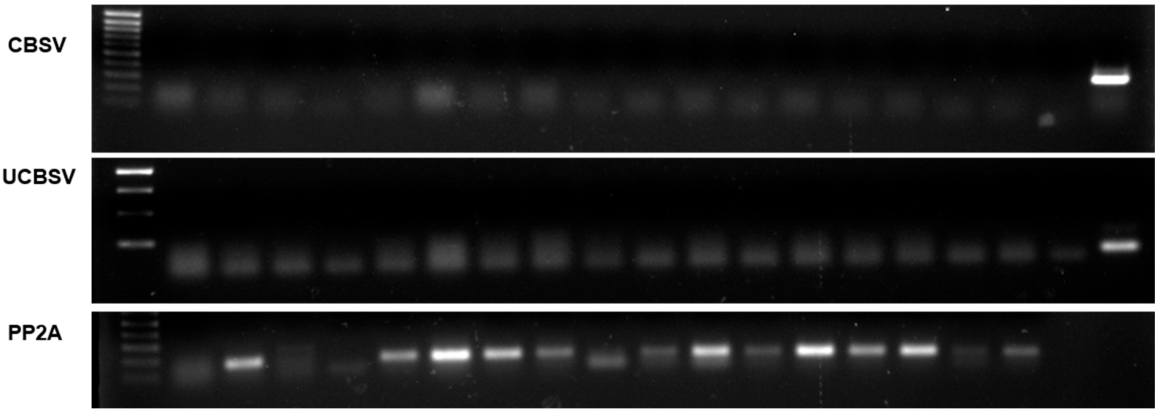

Figure S2. PCR screening for CBSV and UCSBV in selected field-grown cassava cultivars in Ghana and leaf samples from farmer fields.

Lane M: Gene ruler 1kb DNA ladder, lanes 1-11: selected Ghanaian cassava cultivars (see Materials and Methods), lanes 12-17: examples of leaf material collected from farmer fields in Eastern Region (1 sample group), Greater Accra Region (1 sample group), Central Region (2 sample groups), Ashanti Region (2 sample groups) (see Figure S1), lane 18: water negative control, lane 19 positive control for CBSV (upper panel), UCBSV (center panel) and cassava gene PP2A used as internal control for each sample (lower panel). Primers were used for detection of CBSV (CP3 F/R) and UCBSV (UCBSVF/UCBSDR) isolates within three main phylogenetic clades (see Table S1). The figure is composed of two individual gels stacked as panels.

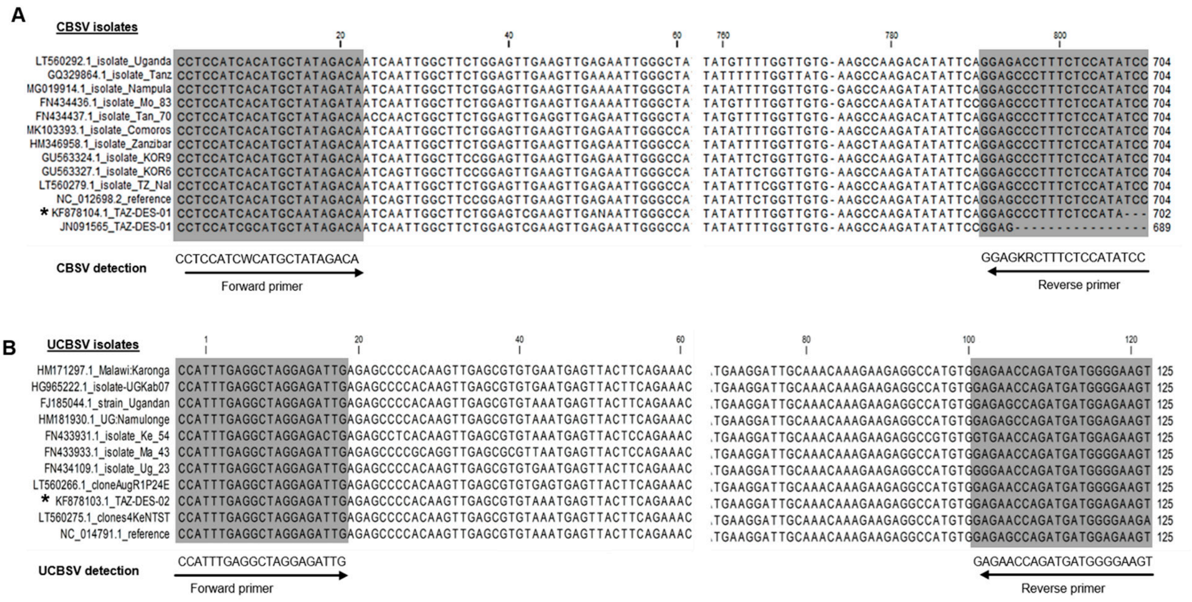

Figure S3. Homology of primers used for RT-PCR screening of cassava cultivars and leaf material collected from farmer fields in Ghana.

(A) Homology of CP3 F/R primers to published CBSV isolates and **(B)** Homology of UCBSVF/R primers to published UCBSV isolates on NCBI database. CBSV (TAZ-DES-01) and UCBSV (TAZ-DES-02) isolates indicated by asterisk symbol. Sequence alignment was carried out with CLC Genomics Workbench, v.10.

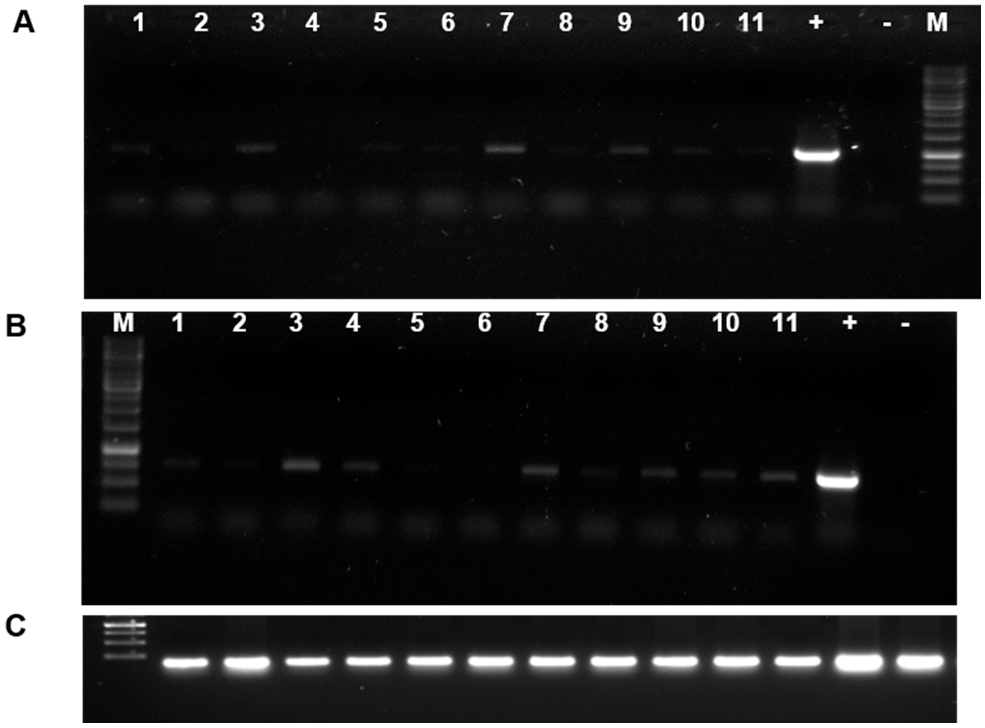

Figure S4. PCR screening of eleven selected cassava cultivars grown in Ghana for cassava mosaic geminiviruses.

(**A**) Use of primers UVAL1/F - ARO/R primers [1] (**B**) primers JSP 001 / 002 primers [2-3] for detection of ACMV and EACMV species in Ghanaian cassava cultivars and (**C**) cassava gene PP2A used as internal control for each sample. Figure is composed of two individual gels stacked as panels.

Table S2. Top-cleft graft transmission of CBSV and UCBSV to farmer-preferred Ghanaian cassava cultivars at 12 weeks

Genotype	Status	CMD response	Number infected / number grafted (%)	Mean CBSD leaf symptom severity
KBH 2006/18	Breeding line	Moderate	0 / 3 (0)	1.0
Ebwanateraka	Released variety	Susceptible	3 / 3 (100)	4.3
60444	Cultivar	Susceptible	3 / 3 (100)	4.3
ADI 001	Landrace	Moderate	3 / 3 (100)	2.3
Afisiafi	Released variety	Resistant	3 / 3 (100)	2.0
Ankra	Landrace	Moderate	3 / 3 (100)	3.7
Bosomnsia	Landrace	Susceptible	3 / 3 (100)	3.7
Dagarti	Released variety	Moderate	3 / 3 (100)	4.0
IFAD	Released variety	Susceptible	3 / 3 (100)	4.3
Megyewontem	Landrace	Susceptible	3 / 3 (100)	4.0
Nkabom	Released variety	Susceptible	3 / 3 (100)	3.0
Santum	Released variety	Moderate	3 / 3 (100)	2.3
Tomfa	Released variety	Susceptible	3 / 3 (100)	3.0
Tuaka	Landrace	Susceptible	3 / 3 (100)	3.0

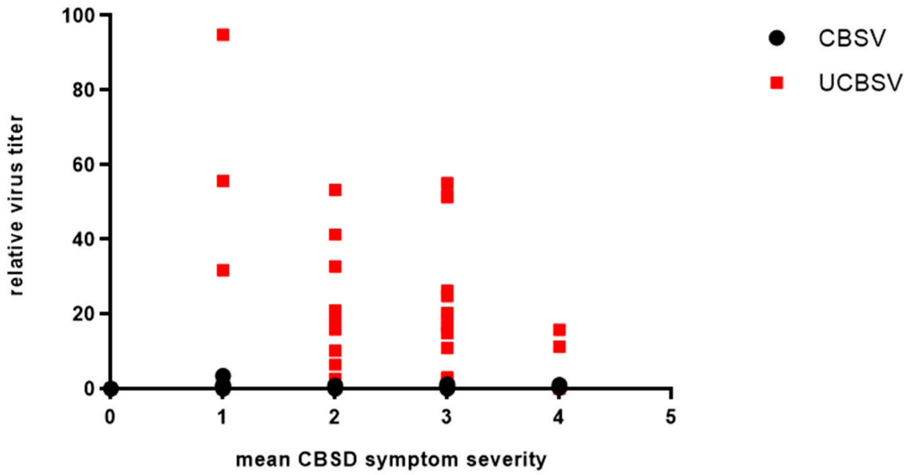

Figure S5. Pearson correlation between CBSD symptom severity in leaves and relative CBSV titers in leaves of Ghanaian cassava genotypes. Symptom severity does not correlate significantly with relative titers of CBSVs (P= 0.9).

Table S3A. Two-way ANOVA comparing CBSV titers in parental scions and cuttings propagated from these scions of Ghanaian cassava cultivars

Source of Variation	% of total variation	P value	P value summary	Significance
Interaction	28,67	0,0174	*	Yes
Cultivars	15,03	0,2909	ns	No
Treatment	3,196	0,0827	ns	No

ANOVA table	SS	DF	MS	F (DFn, DFd)	P value
Interaction	12,51	12	1,043	F (12, 52) = 2,34	P=0,0174
Cultivars	6,559	12	0,5466	F (12, 52) = 1,227	P=0,2909
Treatment	1,395	1	1,395	F (1, 52) = 3,13	P=0,0827
Residual	23,17	52	0,4456		

SS = sum of squares; DF= degrees of freedom; MS = mean squares; F (DFn, DFd) = F ratio (MS value divided by residual)

Table S3B. Šidák's multiple comparisons test of CBSV titers in parental scions and cuttings propagated from scions of Ghanaian cassava cultivars

CBSV parental scions - CBSV propagated cuttings	Mean Difference	95,00% CI of difference	Significance	Adjusted P Value
KBH18	0,002556	-1,642 to 1,647	No	>0,9999
60444	-0,8888	-2,534 to 0,7559	No	0,7769
ADI 001	-1,108	-2,753 to 0,5364	No	0,4660
AFISIAFI	1,496	-0,1489 to 3,141	No	0,1027
ANKRA	-0,1779	-1,823 to 1,467	No	>0,9999
BOSO	-1,741	-3,386 to -0,09661	Yes	0,0305
DAGARTI	0,4719	-1,173 to 2,117	No	0,9984
IFAD	-0,01115	-1,656 to 1,634	No	>0,9999
MEGYE	0,3906	-1,254 to 2,035	No	0,9998
NKABOM	-0,2852	-1,93 to 1,36	No	>0,9999
SANTUM	-0,5116	-2,156 to 1,133	No	0,9965
TOMFA	-1,11	-2,755 to 0,5349	No	0,4639
TUAKA	-0,00326	-1,648 to 1,642	No	>0,9999

Table S4A. Two-way ANOVA comparing titers of UCBSV in parental scions and cuttings propagated from scions of Ghanaian cassava cultivars

Source of Variation	% of total variation	P value	P value summary	Significance
Interaction	23,31	0,0006	***	Yes
cultivars	38,65	<0,0001	****	Yes
Column Factor	10,11	<0,0001	****	Yes

ANOVA table	SS	DF	MS	F (DFn, DFd)	P value
Interaction	3288	12	274	F (12, 52) = 3,618	P=0,0006
cultivars	5451	12	454,2	F (12, 52) = 5,997	P<0,0001
Column Factor	1426	1	1426	F (1, 52) = 18,83	P<0,0001
Residual	3938	52	75,74		

SS = sum of squares; DF= degrees of freedom; MS = mean squares; F (DFn, DFd) = F ratio (MS value divided by residual

Table S4B. Šidák's multiple comparisons test of UCBSV titers in parental scions and cuttings propagated from scions of Ghanaian cassava cultivars

UCBSV parental scions - UCBSV propagated cuttings	Mean Difference	95,00% CI of diff,	Significance	Adjusted P Value
KBH18	0,02236	-21,42 to 21,47	No	>0,9999
60444	-2,956	-24,4 to 18,49	No	>0,9999
ADI 001	30,41	8,969 to 51,86	Yes	0,0010
AFISIAFI	0,001204	-21,44 to 21,44	No	>0,9999
ANKRA	25,02	3,575 to 46,46	Yes	0,0117
BOSO	-1,048	-22,49 to 20,4	No	>0,9999
DAGARTI	17,77	-3,675 to 39,21	No	0,1848
IFAD	15,09	-6,357 to 36,53	No	0,4000
MEGYE	7,388	-14,05 to 28,83	No	0,9909
NKABOM	30,56	9,116 to 52	Yes	0,0010
SANTUM	-4,765	-26,21 to 16,68	No	0,9999
TOMFA	-5,097	-26,54 to 16,35	No	0,9998
TUAKA	-1,227	-22,67 to 20,22	No	>0,9999

Table S5A. Two-way ANOVA comparing survival rate of cuttings propagated from CBSD-infected Ghanaian cassava cultivars at 12 weeks old

Source of Variation	% of total variation	P value	P value summary	Significance
cultivars	11.37	0.6440	ns	No
CBSD infection	74.62	<0.0001	****	Yes

ANOVA table	SS	DF	MS	F (DFn, DFd)	P value
cultivars	3979	13	306.0	F (13, 13) = 0.8114	P=0.6440
CBSD infection	26108	1	26108	F (1, 13) = 69.22	P<0.0001
Residual	4903	13	377.2		

SS = sum of squares; DF= degrees of freedom; MS = mean squares; F (DFn, DFd) = F ratio (MS value divided by residual

Table S5B. Sidak's multiple comparisons test of survival rates of cuttings propagated from CBSD-infected Ghanaian cassava cultivars at 12 weeks old

Control – CBSD infected cuttings	Mean Difference	95,00% CI of difference	Significance	Adjusted P Value
60444	61.07	35.12 to 87.02	Yes	<0.0001
KBH18	61.07	35.12 to 87.02	Yes	<0.0001
EBWANA	61.07	35.12 to 87.02	Yes	<0.0001
ADI 001	61.07	35.12 to 87.02	Yes	<0.0001
AFISIAFI	61.07	35.12 to 87.02	Yes	<0.0001
ANKRA	61.07	35.12 to 87.02	Yes	<0.0001
BOSO	61.07	35.12 to 87.02	Yes	<0.0001
DAGARTI	61.07	35.12 to 87.02	Yes	<0.0001
IFAD	61.07	35.12 to 87.02	Yes	<0.0001
MEGYE	61.07	35.12 to 87.02	Yes	<0.0001
NKABOM	61.07	35.12 to 87.02	Yes	<0.0001
SANTUM	61.07	35.12 to 87.02	Yes	<0.0001
TOMFA	61.07	35.12 to 87.02	Yes	<0.0001

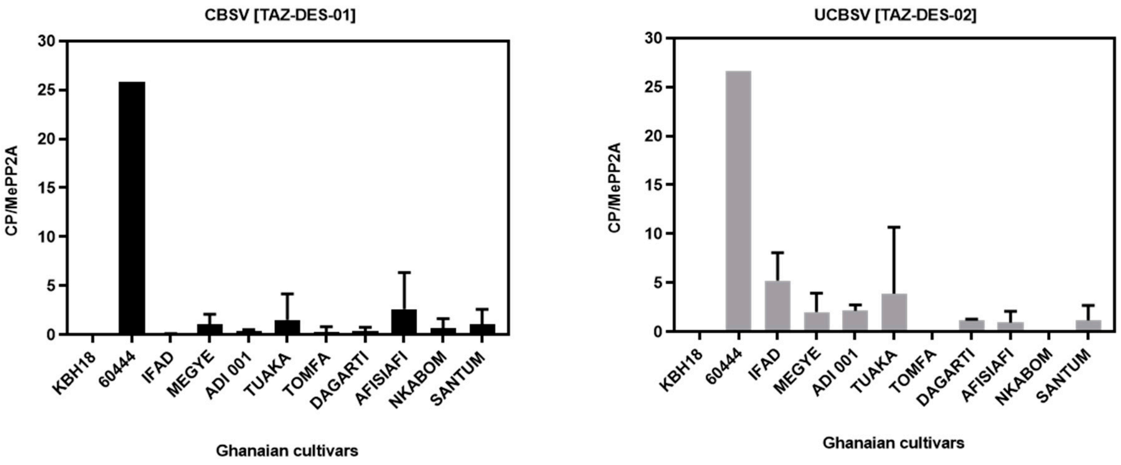

Figure S6. RT-qPCR quantitation of CBSV and UCBSV in roots of Ghanaian cassava genotypes (6 month-old plants)

References

1. Zhou, X.; Liu, Y.; Calvert, L.; Munoz, C.; Otim-Nape, G. W.; Robinson, D. J.; Harrison, B. D., Evidence that DNA-A of a geminivirus associated with severe cassava mosaic disease in Uganda has arisen by interspecific recombination. *J Gen Virol* **1997,** *78 (Pt 8)*, 2101-11.

2. Fondong, V. N.; Pita, J. S.; Rey, M. E.; de Kochko, A.; Beachy, R. N.; Fauquet, C. M., Evidence of synergism between African cassava mosaic virus and a new double-recombinant geminivirus infecting cassava in Cameroon. *J Gen Virol* **2000,** *81* (Pt 1), 287-97.

3. Pita, J. S.; Fondong, V. N.; Sangaré, A.; Otim-Nape, G. W.; Ogwal, S.; Fauquet, C. M., Recombination, pseudorecombination and synergism of geminiviruses are determinant keys to the epidemic of severe cassava mosaic disease in Uganda. *Journal of General Virology* **2001,** *82* (3), 655-665.
